# Combatting Pseudoscience: A Science and Health Literacy Workshop to Improve Scientific Literacy in 16-Year-Old Students in Malaysia

**DOI:** 10.21315/mjms2019.26.5.1

**Published:** 2019-11-04

**Authors:** Hannah M Nazri

**Affiliations:** Nuffield Department of Women’s & Reproductive Health, University of Oxford, Women’s Centre, John Radcliffe Hospital, United Kingdom

**Keywords:** critical thinking, education, health literacy, health advocacy, misinformation, Malaysia

## Abstract

The rise of dubious medical practice and anti-vaccination groups in Malaysia suggests that the public needs to be equipped with the scientific literacy skills to navigate the healthcare landscape. Additionally, the overall result of the Programme for International Student Assessment (PISA) 2009+ for Malaysia suggests that the national scientific literacy levels of 16-year-old Malaysian students to be below the international and the Organisation for Economic Co-operation and Development (OECD) average. Consequently, the Higher Order Thinking Skills (HOTS) was introduced to form part of the national English language evaluation in 2013 to encourage creative and critical thinking. In this editorial piece, I describe a youth-led intervention that may be more effective at increasing scientific literacy to combat pseudoscience in Malaysian youth especially in bridging the education inequality gap in Malaysia.

## Introduction

The advent of complementary and alternative medicine (1), the growth of anti-vaccination groups (2, 3), and regulation failure of cosmetic products suggest that the public needs to be equipped with scientific literacy skills to navigate the Malaysian healthcare landscape especially when some of these pseudoscience practices are advocated by medical professionals. Who is the expert? A medical doctor, unless a specialist, may not be able to comment on stem cell therapy, for example. A stem-cell researcher can provide insight into the therapeutic potentials of stem cells but cannot comment on its direct clinical applications. Stem cell therapy, especially tissue and cord blood stem cells, are most established in blood and immune disorders (4). Consequently, clinics or products claiming to use stem cells other than for blood or immune disorders such as cosmetics are potentially promoting ineffective and unsafe science.

**Figure 1:**
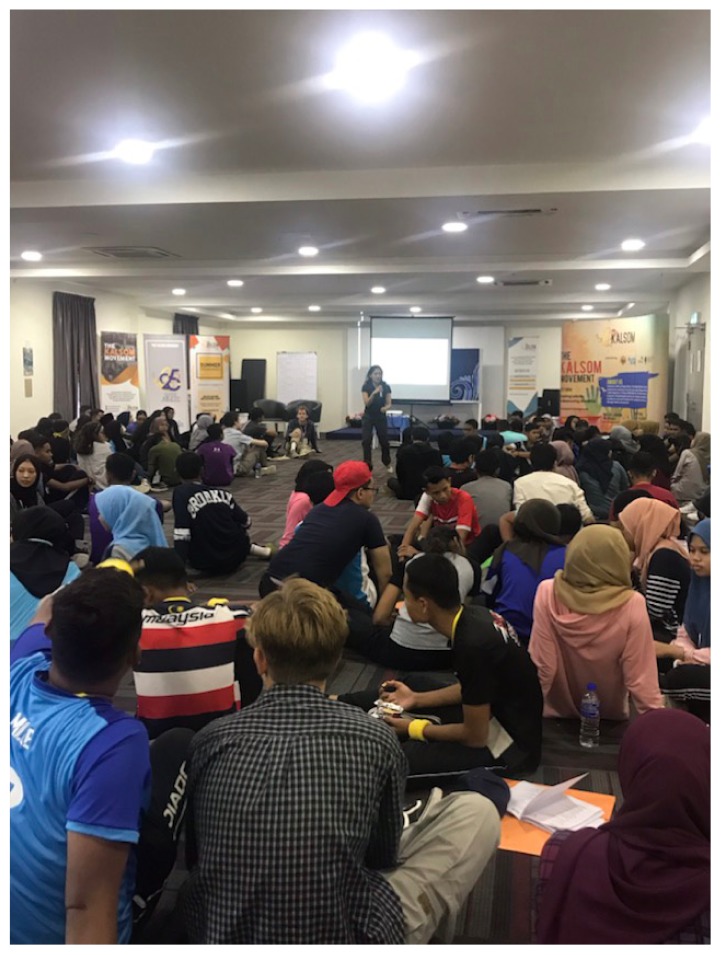
The science and health literacy workshop in session

**Figure 2:**
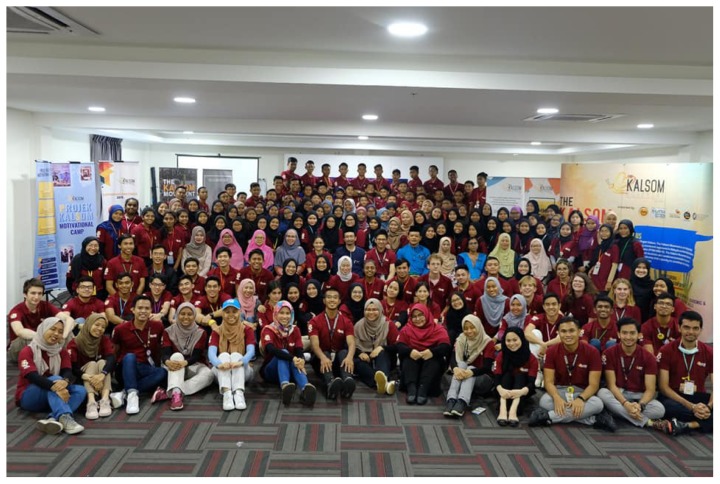
The 25th Projek Kalsom Motivational Camp 2019 cohort of facilitators and students

These were some of the issues discussed during a science and health literacy workshop for 16-year-old students during the 25th Projek Kalsom Motivational Camp (PKMC) in Kota Tinggi in July 2019 (Figure 1). Clearly, an understanding of what constitutes an expert is crucial to separate facts from myths. PKMC is one of the programmes organised by the Kalsom Movement (5), a student-led education charity that empowers university students to mentor younger disadvantaged Malaysian secondary school students (Figure 2).

The idea for this workshop was first conceived during the 2018 Asia-Europe Foundation Young Leaders’ Summit, Brussels as part of the Science & Technology group outcomes as a possible youth-led intervention to combat pseudoscience through increasing scientific literacy. The Programme for International Student Assessment (PISA) defines scientific literacy as the ability ‘to understand the characteristics of science and the significance of science in our modern world, to apply scientific knowledge, identify issues, describe scientific phenomena, draw conclusions based on evidence, and the willingness to reflect on and engage with scientific ideas and subjects’ (6). Worryingly, almost 60% of 15-year-old Malaysian students failed the PISA minimum requirement for reading, mathematics and science, with Malaysia’s overall PISA 2009+ results in the ‘bottom third of 74 participating countries, below the international and the Organisation for Economic Co-operation and Development (OECD) average’ (7). Thus, the 2001 Bloom’s Taxonomy Higher Order Thinking Skills (HOTS) (8) was introduced in English language evaluation in Malaysia in 2013 (7) in a bid to encourage critical thinking and ultimately to develop scientific leaders.

An increasingly encouraged method for combatting scientific misinformation is one that is led by medical professionals and scientists on social media platforms (9) either in their personal capacities or informal social media collaboratives by becoming the ‘nerd of trust’ in their social networks (10), or as part of an institution (11). However, the premise of this workshop is to encourage healthy scepticism over the vast amount of information that has been fed to us in this Internet age, by utilising case studies (12) that mirror real life situations, rather than to teach the students scientific facts or to exhaust them with epistemology. The objectives were set:

- To encourage students to be creative thinkers who question and challenge the information that is presented to them.
- To train students to develop the scientific method in researching and analysing information.

## Methods and Outcomes

One hundred and six students (40 males, 66 females) were selected from SMK Bandar Mas, SMK Pengerang Utama, SMK Bandar Easter, SMK Adela, SMK Semenchu, SMK Air Tawar and SMK Bandar Kota Tinggi in Johor, Malaysia for PKMC. Parental consent was sought and given for the participating students. Students chosen for PKMC are academically-capable 16-year-old Malaysian students from low performing schools as defined by the Malaysian Ministry of Education (Bands 5–7) (7) and from bottom 40% (13) economic group.

The students were randomly assigned into 10 groups with two university student-mentors per group for the two-hour workshop. The workshop was organised into two interactive parts with three main questions forming the basis of the workshop. The questions are chosen to reflect current local issues and to provide a comprehensive scope for discussion based on the students’ level of education:

- Can water be poison?
- Traditional herbal medicines are better than pharmaceutical drugs. Discuss.
- Is the earth flat or round?

The first part of the workshop began by encouraging the students to give opinions about the validity (or absurdity) of the three questions above. This was followed by a discussion on how these questions can be answered, surmised in this answer toolkit:

- **Asking an expert:** A discussion on what constitutes an expert by introducing real-life scenarios and ways to validate and confirm a person’s qualifications and experiences.
- **Researching online:** A discussion of primary and secondary resources, the highest forms of evidence from the Internet and social media platforms. This also includes determining the expertise of writers of online resources, specific marketing agendas by companies, the validity of product testimonials, and an understanding of product placement and branding.
- **Performing an experiment:** This is to encourage students to perform their own investigations where appropriate and possible. The randomised controlled trial was touched briefly.

The second part of the workshop involved group work to answer the three main questions by utilising the answer toolkit.

- **Can water be poison?** 7/10 groups answered ‘no’ with reasons such as ‘water constitutes about 60% of the body’, ‘water is the source of life’ and ‘if water were poison, many would have died.’ Three groups answered the possibility of ‘yes’ with the following reasons: ‘it depends on the cleanliness of the water’, ‘water can be poison if poison was added to it’ and ‘too much water can cause an overload to the heart and kidney and the kidney cannot cope’—true in cardio-renal syndrome patients. ‘Water toxicity’ was explored by one group.
- **Traditional herbal medicines are better than pharmaceutical drugs. Discuss**. 4/10 groups said ‘yes’, 5/10 said ‘no’ and one group suggested a tie. Groups that said ‘yes’ voiced concerns about the ‘side-effects of pharmaceutical drugs.’ ‘No’ groups voiced concerns about the ‘lack of regulatory mechanisms in herbal products sold’ and that ‘pharmaceutical drugs are safer as they have been 100% tested.’ The ‘tie’ group suggested that ‘both has its good and bad depending on how much you take them.’ Indeed, what makes a drug safe includes a comprehensive safety profile which tends to be more rigorously tested in pharmaceutically-derived drugs. The discovery of the anti-malarial drug, quinine from the cinchona tree and the senna drug, a known natural laxative, were discussed.
- **Is the earth flat or round?** 100% of the students agreed that earth is a sphere by quoting evidence from NASA, the phenomena of day and night and different time zones, and earth curvature experiments.

## Conclusion

The effectiveness of this workshop in combatting pseudoscience is too early to tell, but initial results are promising and further feedback mechanisms such as a science test specifically to test HOTS and in information finding may be employed to gauge improvements in scientific literacy in the future. Students from the rural area of Kota Tinggi, Johor can communicate their opinions on science and health issues in the English language with encouragement and facilitation from peers nearer to their age group (university students). This suggests that regular scientific discussions in a supportive environment such as in PKMC can help students to develop their scientific literacy.
